# Supplementary material for: Identification of photoperiod-induced specific miRNAs in the adrenal glands of Sunite sheep (Ovis aries)
Source: Front Vet Sci. 2022 Jul 22;9:888207. doi: 10.3389/fvets.2022.888207 (PMC9354845; doi:10.3389/fvets.2022.888207)
Supplement: Supplementary file 1 [file Data_Sheet_1.ZIP › Supplementary file/miRDeep2 file.zip!/miRDeep2 file/Novel_Structure/SPLP42A2/Novel_248.pdf]

Provisional ID	:	NC_019472.2_28995
Score total	:	48.6
Score for star read(s)	:	3.9
Score for read counts	:	41.4
Score for mfe	:	1.7
Score for randfold	:	1.6
Score for cons. seed	:	
Total read count	:	93
Mature read count	:	91
Loop read count	:	0
Star read count	:	2

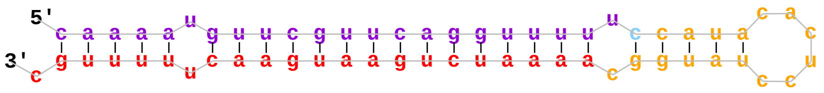

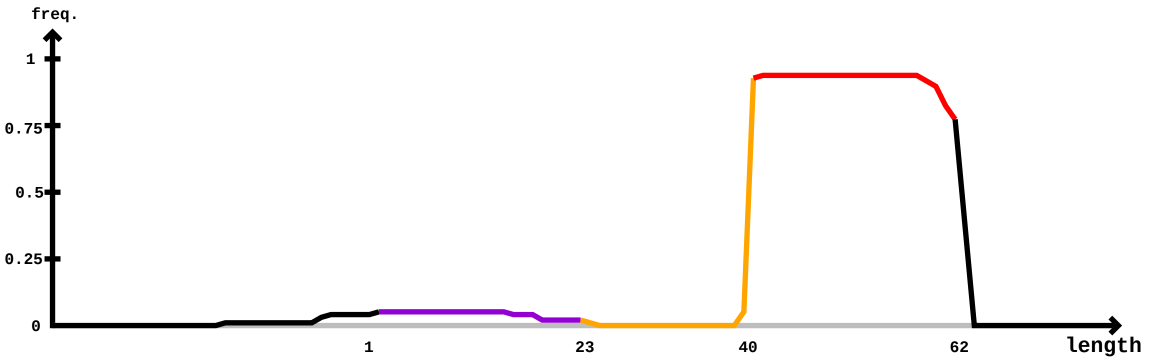

Star **Mature**

```
5'- ugaagcacaaaaagcuuauguauuggguuggccaaaauguucguucagguuuuuccauacacuccuauggcaaaaucugaaugaacuuuuugcccauuugaacucaauc   -3'    obs
    ugaagcacaaaaagcuuauguauuggguuggccaaaauguucguucagguuuuuccauacacuccuauggcaaaaucugaaugaacuuuuugcccauuugaacucaauc          exp
    ..((((.......))))....(((((((((.(((((.(((((((((((((((.(((((......))))).))))))))))))))).))))).))).....))))))).   reads     mm        sample
    ................uauguauuggguuggcc............................................................................   1         0         seq
    ..........................guuggccaaaauguucguuU...............................................................   1         1         seq
    ..........................guuggccaaaauguucguucagU............................................................   1         1         seq
    ...........................uuggccaaaauguucguucagg............................................................   1         0         seq
    ................................caaaaAguucguucagguuuuu.......................................................   1         1         seq
    .................................aaaaAguucguucagguuuuuc......................................................   1         1         seq
    ......................................................................caaaaucugaaugaacuuu....................   2         0         seq
    ......................................................................caaaaucugaaugaacuuuu...................   1         0         seq
    ......................................................................caaaaCcugaaugaacuuuuu..................   1         1         seq
    ......................................................................caaaaucugaaugaacuuuuu..................   1         0         seq
    .......................................................................aaaCucugaaugaacuuuu...................   1         1         seq
    .......................................................................aaaaucuAaaugaacuuuuu..................   2         1         seq
    .......................................................................aaaaucugaauAaacuuuuu..................   1         1         seq
    .......................................................................aaaaucugaaugaacCuuuu..................   1         1         seq
    .......................................................................aaaCucugaaugaacuuuuug.................   4         1         seq
    .......................................................................aaaaucugaaugaacuuuuuC.................   1         1         seq
    .......................................................................aaaaucugaaugaacuuuuGgc................   3         1         seq
    .......................................................................aaaaucugaaugaacuuuuugc................   6         0         seq
    .......................................................................aaaaucugaaugaacuuuuugA................   66        1         seq
    ........................................................................aaaucugaaugaacuuuuu..................   1         0         seq
```
